# Assessing the Fates of Water and Nitrogen on an Open-Field Intensive Vegetable System under an Expert-N System with EU-Rotate_N Model in North China Plain

**DOI:** 10.3390/plants13152150

**Published:** 2024-08-03

**Authors:** Yuan Sun, Shaoqing Chen, Puyu Feng, Qing Chen, Kelin Hu

**Affiliations:** 1 College of Land Science and Technology, China Agricultural University, Key Laboratory of Arable Land Conservation (North China), Ministry of Agriculture and Rural Affairs, Beijing 100193, China; sunyuan01@caas.cn (Y.S.); b20223211022@cau.edu.cn (S.C.); fengpuyu@cau.edu.cn (P.F.); 2 Agricultural Information Institute of Chinese Academy of Agricultural Science, Beijing 100081, China; 3 College of Resources and Environmental Sciences, China Agricultural University, Beijing 100193, China; qchen@cau.edu.cn

**Keywords:** open-field vegetable, water drainage, nitrate leaching, vegetable yield

## Abstract

Nitrate leaching, greenhouse gas emissions, and water loss are caused by conventional water and fertilizer management in vegetable fields. The Expert-N system is a useful tool for recommending the optimal nitrogen (N) fertilizer for vegetable cultivation. To clarify the fates of water and N in vegetable fields, an open-field vegetable cultivation experiment was conducted in Dongbeiwang, Beijing. This experiment tested two irrigation treatments (W1: conventional and W2: optimal) and three fertilizer treatments (N1: conventional, N2: optimal N rate by Expert-N system, and N3: 80% optimal N rate) on cauliflower (*Brassica oleracea* L.), amaranth (*Amaranthus tricolor* L.), and spinach (*Spinacia oleracea* L.). The EU-Rotate_N model was used to simulate the fates of water and N in the soil. The results indicated that the yields of amaranth and spinach showed no significant differences among all the treatments in 2000 and 2001. However, cauliflower yield under the W1N2 and W1N3 treatments obviously reduced in 2001. Compared with the W1 treatment, W2 reduced irrigation amount by 27.9–29.8%, water drainage by over 76%, increased water use efficiency by 5–17%, and irrigation water use efficiency by 29–45%. Nitrate leaching was one of the main pathways in this study, accounting for 8.4% of the total N input; compared to N1, the input of fertilizer N under the N2 and N3 treatments decreased by over 66.5%, consequently reducing gaseous N by 48–72% and increasing nitrogen use efficiency (NUE) by 17–37%. Additionally, compared with the W1 treatments, gaseous N loss under the W2 treatments was reduced by 18–26% and annual average NUEs increased by 22–29%. The highest annual average NUEs were under W2N3 (169.6 kg kg^−1^) in 2000 and W2N2 (188.0 kg kg^−1^) in 2001, respectively. We found that optimizing fertilizer management allowed subsequent crops to utilize residual N in the soil. Therefore, we suggest that the W2N3 management should be recommended to farmers to reduce water and N loss in vegetable production systems.

## 1. Introduction

Vegetable crops are high-priced cash crops with short growing periods and high returns. They require large amounts of water and nitrogen (N) to grow rapidly and attain high yields [1]. Therefore, vegetable fields are particularly prone to water loss and N pollution [2,3]. The North China Plain (NCP) serves as a significant center for vegetable cultivation in China, where the shortage of water resources becomes the primary constraint. Previous research have proposed that conventional irrigation and N fertilizer practices lead to lower nitrogen use efficiency (NUE) and higher residual N in the soil profile in this area [4,5,6]. Furthermore, with the increasing areas of vegetable cultivation, the less-developed root systems of vegetable crops have caused large amounts of nitrate leaching, and resulted in the contamination of groundwater [7,8].

To obtain the best management practices for intensive vegetable systems, many water and N fertilizer practices were applied to improve water use efficiency (WUE) and NUE. Compared with conventional management, optimized N application rates significantly decreased the nitrate leaching loss by 47% without negative impacts on N uptake and tomato yields [8]. A meta-analysis revealed that reducing the input rates of N fertilizers or irrigation water brought advantages for maintaining vegetable yields, increasing NUE, decreasing N_2_O emissions, and reducing reactive N released into the environment [9]. Another vegetable crop rotation experiment indicated that a combination of drip irrigation and high irrigation water input could achieve a balance of high yields and low environmental risks [10]. N expert systems, such as the KNS system (Kulturbegleitende Nmin Software V4.6) are useful tools for managing N fertilizer applications in vegetable fields and have been widely used in Europe [11,12]. Later, a modified N-Expert system for vegetable cultivation was proposed by Chen et al. [13], and results indicated that this N recommendation system could reduce N fertilizer input and N residual in soil in the NCP. However, the fate of N under the N-Expert system remains unclear.

After the popularization of computers, human knowledge of agricultural systems was captured in computer models to guide farmers in making decisions on the different aspects of crop management [14]. In most cases, mechanistic models perform well in simulating the process of nitrate leaching, crop N uptake, and crop production quality [15,16,17]. For example, the NLEAP model was developed to estimate the potential of nitrate leaching in crop fields [18,19,20]. Alternatively, based on the N_ABLE model, the EU-Rotate_N model is a more powerful tool for simulating water and N dynamics in soil/plant systems [21]. The EU-Rotate_N model has been successfully used to simulate water and N dynamics and vegetable growth in some studies in Europe and China [22,23,24,25]. Their results suggested that the EU-Rotate_N model worked well in tracking water and N fates under different irrigation and fertilizer management. However, few studies have focused on the application of the EU-Rotate_N model for various continuous vegetable cultivation systems.

Open-field vegetable cultivation is one of the main vegetable-planting patterns in the NCP. The issues of water drainage, nitrate leaching, and gaseous N release in greenhouse vegetable planting systems have already been extensively explored in previous works [8,23,26,27], but those processes are more complex and less certain under open-field vegetable cultivation systems. Moreover, in the NCP, three varieties of vegetables are typically cultivated consecutively within a single year, leading to an increased frequency of water and N applications to the soil. Based on the above studies, we proposed the following hypotheses: (i) the improved N expert systems could save more water and fertilizer resources and (ii) optimized water and N management practices will significantly reduce water drainage and N loss, and improve WUE and NUE compared to the conventional practices in the NCP.

Therefore, our aims are (i) to calibrate and validate the EU-Rotate_N model with water and soil mineral N contents measured under the conventional and N-Expert systems on an open-field vegetable-growing system and (ii) to assess the fates of water and N, and WUE and NUE under conventional and optimized water and N management in an intensive vegetable system.

## 2. Results

### 2.1. Model Calibration and Validation

**Figure 1:**
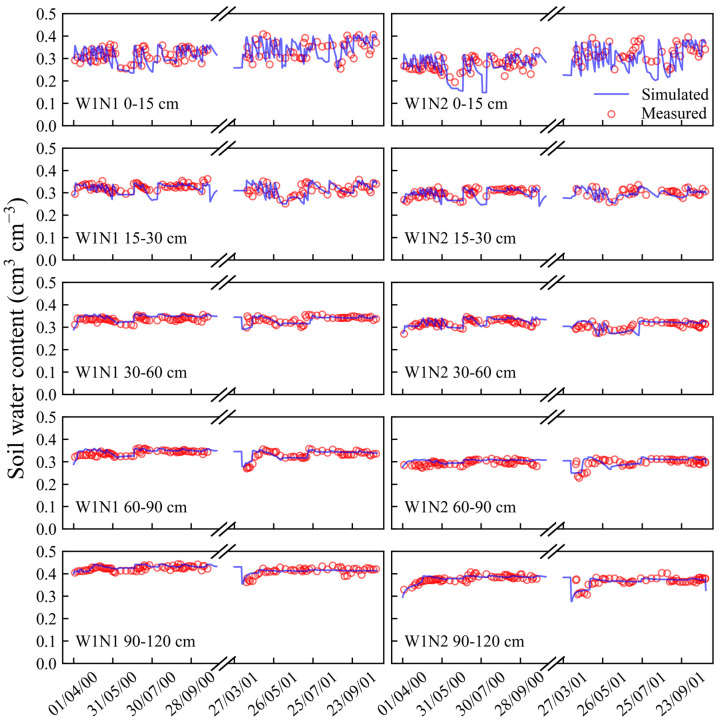
Comparisons of simulated and measured soil water content in 0–120 cm soil profile for W1N1 and W1N2 treatments in 2000 and 2001.

The soil water content, soil nitrate concentration, dry weight, N uptake, and yields of vegetables collected from the W1N1 treatment were used to calibrate the EU-Rotate_N model. The irrigation and N fertilizer applications, as detailed in Table S1, were incorporated into the model. Calibration was achieved through a ‘trial and error’ approach, adjusting parameters to align the simulated outcomes with the measured data [8,27,28,29]. The calibration was processed in the following order: soil water content, nitrate concentration, crop N uptake, vegetable yield, and crop dry matter [23,30]. Crop coefficients and luxury N consumption coefficients for the three vegetables were adjusted based on measured vegetable yield and N uptake (Table S2). Adjustments to N transformation parameters were guided by the comparison of the simulated and actual soil nitrate concentrations. The modified parameter values are listed in Table S2. Additionally, Figure 1 and Figure S2 compare the simulated and actual measurements of the soil water content, soil nitrate concentration, and dry matter weight for the W1N1 treatment, demonstrating a commendable correlation between the simulated and observed values.

**Figure 2:**
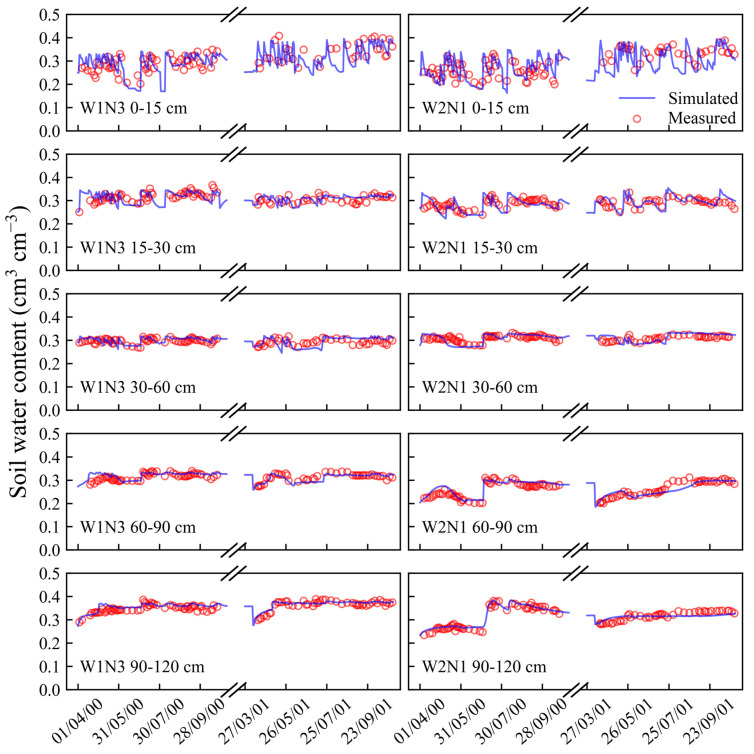
Comparisons of simulated and measured soil water content in 0–120 cm soil profile for W1N3 and W2N1 treatments in 2000 and 2001.

**Figure 3:**
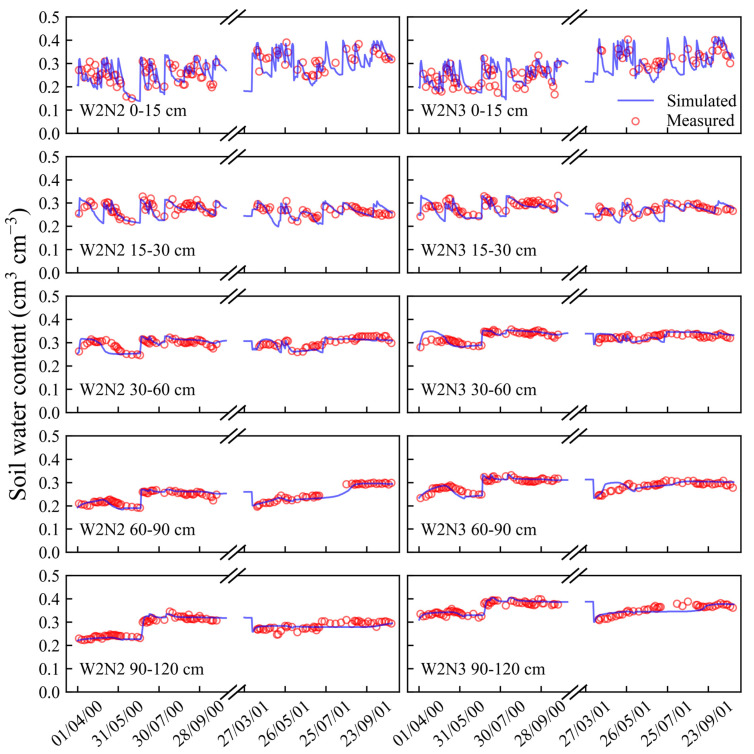
Comparisons of simulated and measured soil water content in 0–120 cm soil profile for W2N2 and W2N3 treatments in 2000 and 2001.

**Figure 4:**
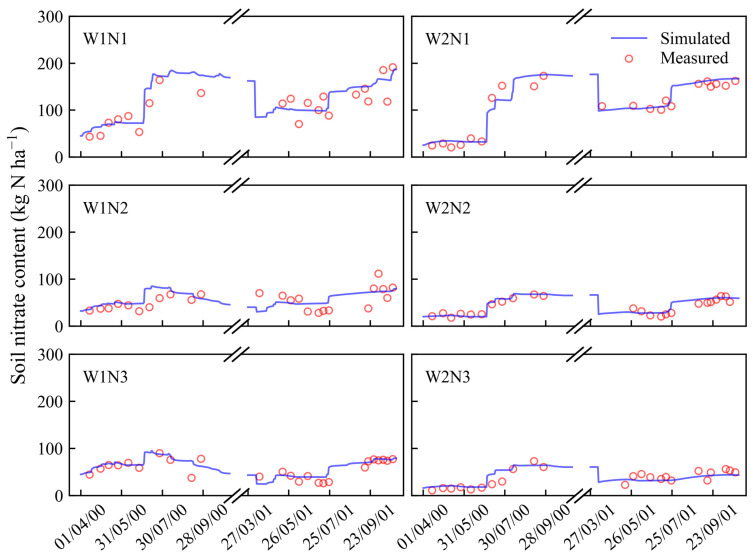
Comparisons of simulated and measured soil nitrate concentration at 60–90 cm soil layer under different treatments in 2000 and 2001.

After the calibration, the model was validated by the datasets collected from the other treatments (W1N2, W1N3, W2N1, W2N2, and W2N3). The comparisons of the simulated and measured soil water content and nitrate concentrations at different soil layers for all the validation treatments are presented in Figure 1, Figure 2, Figure 3 and Figure 4. Moreover, statistical indices for the simulated soil water content and nitrate concentration for these treatments are illustrated in Table 1. 

As shown in Figure 1, Figure 2 and Figure 3, the surface soil layer (0–15 cm) exhibited frequent fluctuations in the water content, leading to elevated root mean square error (RMSE) values (Table 1). However, the RMSE values for the subsequent soil layers ranged between 0.01 and 0.02. Across all the soil layers, the values of Nash/Sutcliffe efficiency coefficient (E) were more than 0.36, and the values of d ranged from 0.83 to 0.98. These indices suggest a strong concordance between the simulated soil water content and the actual observations [31,32]. As shown in Figure 4, most of the simulated values of the soil nitrate concentration were in good agreement with the observed values. Furthermore, the values of E and agreement index (d) were both above 0.87, indicating a high level of accuracy in the simulation results.

**Figure 5:**
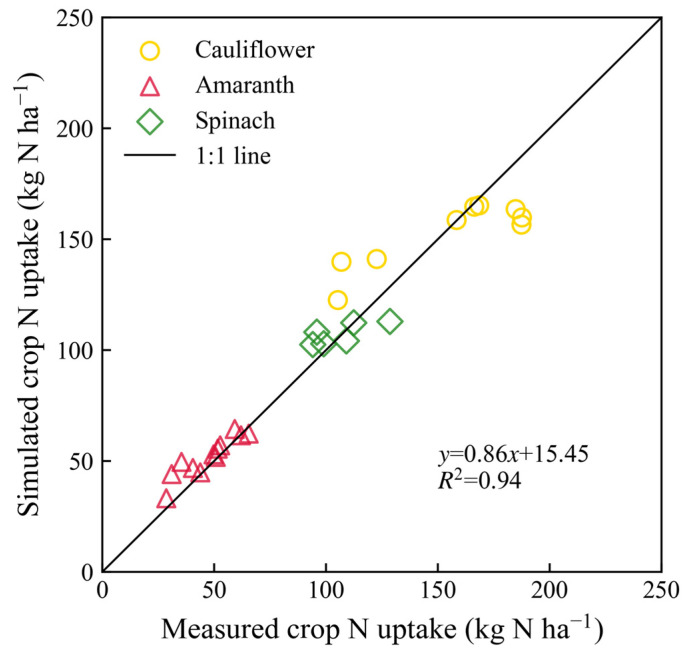
Relationships of simulated and measured crop N uptake for three vegetables (cauliflower, amaranth, and spinach) under all treatments in 2000 and 2001.

The correlation coefficient between the simulated and measured crop N uptake was 0.965, and the *p*-value was significant (*p* < 0.001) (Figure 5). The results shown in Figures S1 and S2 indicate that the simulated crop dry matter weight agreed well with the measured values.

In conclusion, the EU-Rotate_N model performed well in predicting the soil water content, soil nitrate concentration, N uptake, and vegetable growth in this study. Therefore, the model could be used to predict the fate of water and N in vegetable production systems [25,33].

### 2.2. Vegetable Yield, Water Balance and Water Use Efficiency

Different irrigation and fertilizer practices did not result in significant differences in vegetable yield in 2000 and 2001, except for cauliflower (*p* < 0.05, Table 2 and Table 3). The average yield for three vegetables in 2000 and 2001 is as follows: cauliflower, 20,450 and 17,800 kg ha^−1^; amaranth, 13,933 and 19,700 kg ha^−1^; and spinach, 33,933 and 29,933 kg ha^−1^. Over the two years, the W1N1 treatment produced the highest vegetable yield.

The water balance within the 0.9 m soil profile across all the treatments for the years 2000 and 2001 is shown in Table 2 and Table 3. Rainfall and irrigation were the major water resources in this experiment. Notably, the precipitation in 2000 was higher than that in 2001, with the majority occurring during the amaranth-growing season. The total water inputs were as follows: for treatment W1, 769.5 mm in 2000 and 665.2 mm in 2001; for treatment W2, 627.8 mm in 2000 and 509.9 mm in 2001. Evapotranspiration (ET) was the predominant form of water consumption for all the treatments, accounting for 70.5% to 96.2% of the total water input over the two-year period. The ET difference between the different irrigation treatments was so obvious, but under the same irrigation treatments, the difference between different fertilization treatments was very small. Optimizing water input was found to significantly reduce ET within identical fertilizer treatments, although varying fertilizer regimes induced minimal alterations in ET under consistent irrigation management. For instance, in 2000, the total ET under the W1 treatment ranged from 542.7 to 562.0 mm, which decreased to a range of 489.0 mm to 501.3 mm under treatment W2. Among the three vegetables, the order of ET in 2000 was as follows: cauliflower > spinach > amaranth, while the ranking in 2001 was cauliflower > amaranth > spinach. The annual ET was primarily influenced by the ET during the cauliflower growth period.

**Figure 6:**
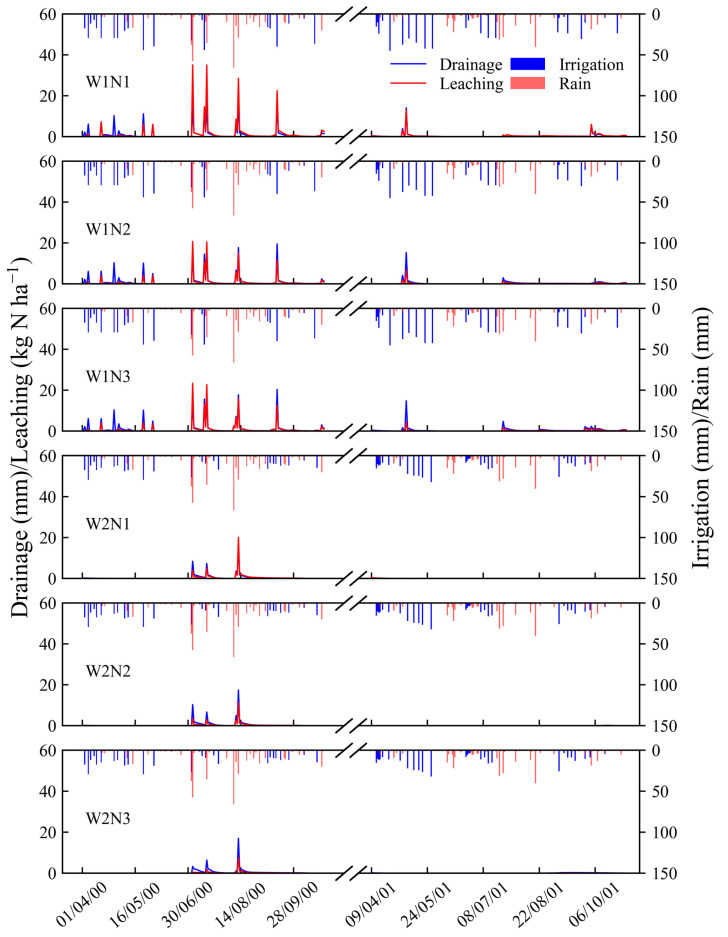
Dynamics of water drainage and nitrate leaching at the 90 cm depth under all of the water and fertilizer treatments in 2000 and 2001.

The simulation results revealed that water drainage accounted for a minor fraction of the total water output in this study. The amount of water drainage was more substantial in 2000 than in 2001, with the highest annual drainage of 168.3 mm and 56.4 mm occurring under the W1N3 treatment in the respective years. Furthermore, the peaks of water drainage mainly occurred after irrigation or heavy rainfall during different vegetable growth seasons in 2000 and 2001, respectively (Figure 6). The study further demonstrated that optimizing irrigation schedules markedly reduced water drainage. The water drainage under the W2 treatment decreased by 74–78% in 2000 and 82–98% in 2001 compared to the W1 treatment, respectively. Moreover, the simulation results indicated that a small quantity of water remained within the 0–0.9 m soil profile after spinach harvest in both years under all the treatments. Conversely, the cauliflower exhibited signs of mild dehydration during the two experimental years.

Regardless of the irrigation and fertilizer management employed, water use efficiencies (WUEs) and irrigation water use efficiencies (IWUEs) in the spinach-growing season were the highest in both two years. However, the WUEs and IWUEs exhibited variable responses among the three vegetable cultivations under differing water and fertilizer regimes. For instance, the highest WUEs of cauliflower were observed under the W2N1 treatment in 2000 and 2001, reaching 7.6 and 7.5 kg m^−3^, respectively. In the case of amaranth, the W2N3 (in 2000) and W2N1 (in 2001) treatments produced optimal WUEs, reaching 16.3 and 19.9 kg m^−3^, respectively. Similarly, spinach achieved its maximum WUEs under the same treatments as those that benefited amaranth, reaching 29.4 and 26.9 kg m^−3^, respectively.

Additionally, the maximum annual WUE was recorded under the W2N2 treatment, while the W2N3 treatment produced the second-highest IWUE in 2000. In 2001, the W2N1 treatment observed the highest annual WUE and IWUE. When considering the same N fertilizer input treatments, there was a negative correlation with irrigation input. For example, the amount of irrigation under the W1N1 treatment increased by 27.9–29.7% compared with W2N1. Consequently, the annual average WUE and IWUE increased by 5–17% and 29–45%, respectively. Under the same irrigation treatments, the patterns of the annual average WUEs and IWUEs in response to the reduced N fertilizer input were ambiguous in 2000. However, the annual average WUEs and IWUEs slightly declined with the reductions in N fertilizer input in 2001. Therefore, alterations in the annual average WUEs and IWUEs were sensitive to irrigation management. 

### 2.3. Nitrogen Balance and Nitrogen Use Efficiency

The N balances in the 0.9 m soil profile for all the treatments in both two years are summarized in Table 4 and Table 5. Besides manure, fertilizer, and irrigation water, the total net mineralization was another source of N, which ranged from 78.0 to 86.5 kg N ha^−1^ in 2000 and 97.0 to 103.1 kg N ha^−1^ in 2001. For all the treatments, most of the N was taken up by the crop, which accounted for 31.7–93.2% and 29.3–81.8% of the total N input in 2000 and 2001, respectively. 

In our research, the results indicated that gaseous N loss (volatilization and denitrification) was the main pathway of N loss in the open-field system under most treatments. The amount of total gaseous N was 53.5–212.9 kg N ha^−1^ in 2000 and 37.5–179.2 kg N ha^−1^ in 2001, accounting for 14.8–23.8% and 9.5–16.3% of the total N input, respectively. The maximum gaseous N release occurred under the W1N1 treatment, reaching up to 212.9 and 179.2 kg N ha^−1^ over the two years. The gaseous N loss varied with the different fertilization and irrigation practices. Under the same irrigation management, optimizing N fertilizer application could effectively reduce gaseous N loss. For example, under the W1 treatment, the N2 and N3 treatments reduced total gaseous N loss by 56–59% and 48–65% by saving 68–73% and 67–80% of the input of N fertilizer compared to the N1 treatment.

The total amount of nitrate leaching ranged from 12.2 to 195.7 kg N ha^−1^ in 2000 and 0.5 to 57.0 kg N ha^−1^ in 2001, respectively. In both years, the W1N1 treatment resulted in the highest total nitrate leaching, accounting for 18.9% and 5.2% of the total N input. In our study, the interaction of both irrigation and fertilizer management had a comparable impact on the total nitrate leaching. For the same fertilizer treatment, reducing irrigation amount significantly decreased the total nitrate leaching in 2000 and 2001. For example, the amount of irrigation under the W2N1 treatment was reduced by 85–97% compared with the W1N1 treatment, which resulted in the amount of total nitrate leaching under the W2N1 treatment being reduced by 166.3 and 55.3 kg N ha^−1^ in 2000 and 2001, respectively. For the same irrigation treatment, total nitrate leaching under W1N2 was reduced by 41–61% compared with W1N1.

The effects of different irrigation and fertilizer management on the amount of soil N residual (N balance) in the 0.9 m soil profile were varied (Table 4 and Table 5). Compared with the conventional fertilizer treatment, optimizing fertilizer treatment could significantly reduce N accumulation in the 0.9 m soil profile under all the irrigation treatments. Especially in 2000, due to the amount and intensity of rainfall, the total soil N residual under N2 and N3 was negative. For the conventional fertilizer treatment (N1), optimizing irrigation increased N residual in the 0.9 m soil profile in 2000 and 2001. The amount of N residual in each vegetable-growing season varied with different irrigation and fertilizer management. Except for the W2N2 treatment in 2000, the N residual in cauliflower season was positive, and was the highest among the three vegetables. This was related to higher N application in this region. During the amaranth-growing season, the N residual was negative under most conditions, especially under the optimizing N fertilizer treatments. 

Our simulation results indicated that the application of N does not exhibit a linear relationship with the N uptake by the vegetables. For example, despite the total N fertilizer application under the W2N2 and W2N3 treatments being lower than the W2N1 treatment in 2000, the N uptake displayed the opposite trend. Moreover, the N uptake of amaranth ranged from 33.2 to 64.4 kg N ha^−1^ under the optimized fertilizer management (Table 4 and Table 5), which was even higher than the application of N in these treatments. These results also suggested that amaranth growth possibly consumed a part of the residual N from the cauliflower season under the optimized fertilizer treatments. For the spinach season, the residual N patterns were similar to those observed in the amaranth season.

The greatest annual average NUEs reached up to 169.6 kg kg^−1^ in 2000 and 188.0 kg kg^−1^ in 2001, which occurred under the W2N3 and W2N2 treatments, respectively (Table 4 and Table 5). The simulation results indicated that for the same irrigation management, the optimizing fertilizer treatments increased annual average NUEs. For example, the annual average NUE under the W1N2 treatment increased by 28% in 2000 and 22% in 2001 compared with the W1N1 treatment. Similarly, the annual average NUE under the W2N2 treatment increased by 29% in 2000 and 20% in 2001 compared with the W2N1 treatment. On the other hand, optimizing irrigation also effectively increased annual average NUEs. For example, the annual average NUE under the W2N1 treatment increased by 29% in 2000 and 22% in 2001, compared with the W1N1 treatment. Moreover, the NUE of each vegetable varied in different years and treatments. In 2000, spinach had the highest NUE among the three vegetables, while in 2001, amaranth did. Over the two years, cauliflower obtained the highest NUE under the W2N2 treatment. For spinach, the W2N3 treatment yielded the maximum NUE. For amaranth, the highest NUE was observed under the W2N3 and W2N2 treatments in 2000 and 2001, respectively.

## 3. Discussion

### 3.1. Water Consumption Responses to Different Water and N Management Practices

Unlike greenhouse vegetable fields, open-field vegetable production relies on both rainfall and irrigation as the main sources of water input, while ET is the main process of water loss. Water drainage only occupies a small fraction of the total water consumption [34,35,36]. As Table 2 and Table 3 show, most of the water was drained during the amaranth-growing season in 2000. However, there was almost no water leakage during this time in 2001. This may be because of the heavy rainfall in 2000 (Figure 6). 

In our research, the EU-Rotate_N model used the Penman/Monteith method of the FAO to simulate ET and the Ritchie water balance equation to calculate water drainage [24,37,38]. ET in the Penman/Monteith method is mainly determined by climatic factors and vegetable cultivar characteristics. As shown in Table 2 and Table 3, the ET values under different N treatments were similar and showed no significant differences, implying that the water uptake capacity of the vegetable crops was not significantly affected by N input. Additionally, ET was strongly correlated with water input in both years of our study, which is consistent with previous studies [39]. 

Water drainage is a major process of water consumption in agricultural systems [40]. It is mainly influenced by soil properties and irrigation practices, and it varies significantly among different water treatments. In particular, under W2 in 2001, the water drainage under each N treatment was significantly lower than the other treatments, and the yield reduction in the vegetable crops was minimal. Our results are consistent with those of Cheng et al. [41], who found that water-saving irrigation can effectively reduce water drainage in vegetable fields without significantly impacting yield. Besides, there are some differences between the different N treatments. In 2001, under W2, the water drainage under N3 was slightly higher than the other N treatments, possibly due to the reduced root development caused by N deficiency, which in turn decreased the water uptake capacity of the vegetables [42].

Climate change will intensify water scarcity, the structural and environmental conditions of open-field vegetable production are more complex than those of greenhouse vegetable production [43,44], and therefore, vegetable management must be adapted to save water [45]. It is necessary to apply greenhouse cultivation techniques for vegetable crops that require sufficient water supply, especially in regions with low rainfall. In addition to optimizing irrigation management and applying greenhouse cultivation techniques, farmers can also adopt drought-tolerant vegetable crops such as amaranth or spinach instead of cauliflower especially in arid areas to reduce irrigation costs and the drought risks.

### 3.2. The Fates of N Responses to Different Water and N Management Practices

In this study, gaseous N loss and nitrate leaching were identified as the main N loss pathways within the open-field vegetable system, with N fertilizer and manure constituting the predominant sources of N input. For all the treatments, crop N uptake was the major N output, which accounted for 31.7–93.2% and 29.3–81.8% of the total N input in 2000 and 2001, respectively. Except for the W2 treatment in 2000, crop N uptake was related positively to N fertilizer rates, which may be caused by greater precipitation in 2000 [7,22]. 

Apart from manure, fertilizer, and irrigation water, total net mineralization was another source of N. Despite the net mineralization of soil N being significantly lower than the other two sources, it may play a critical role for amaranth under the N2 and N3 treatments due to reduced N input. As indicated in Table 4 and Table 5, the total net mineralization observed in our investigation was slightly lower than the values reported by other researchers [25,46]. The variation in the results of our research could be explained by the heterogeneity of soil properties, manure application, and climatic conditions. The simulation results demonstrated that different irrigation practices influenced net mineralization rates, corroborating earlier research that soil moisture and temperature affect mineralization velocity [47]. However, our simulation results indicated that the soil net mineralization rate remained stable across varying fertilization conditions. Notably, different N applications are known to impact net mineralization [48], suggesting that the EU-Rotate_N model requires refinement in its approach to N net mineralization estimation.

Unlike greenhouse vegetable fields, open-field vegetable production had higher gaseous N losses than nitrate leaching, which was the main form of N loss under all the treatments in this study [23,49]. The amount of gaseous N release was the highest under the W1N1 treatment, up to 212.9 and 179.2 kg N ha^−1^ in two years, which was significantly higher than that observed in greenhouse vegetable fields [23,50,51]. Previous studies reported that the amount of gaseous N loss had a positive relationship with N fertilizer rates [23,49,50], which was also proven by our results. On the other hand, optimizing irrigation had a minor effect on reducing gaseous N release. Compared with the W1N1 treatment, the amount of irrigation under the W2N1 treatment was reduced by about 28–30%, and the gaseous N loss declined by 18–26%. This may be because reducing irrigation could decrease gaseous N loss and increase N uptake, as reported by Wang et al. [52]. Under optimized irrigation practices, the concentration of air in the soil remained at a higher level, which inhibited denitrifying bacteria activity. Furthermore, more than 50% of the total N fertilizer was applied in the cauliflower-growing season, which resulted in a higher potential for gaseous N loss.

Many previous studies reported that optimizing irrigation and N fertilizer input is effective in reducing nitrate leaching [8,9,10,22], which is consistent with our results. The contribution of nitrate leaching during each vegetable-growing season to the total nitrate leaching varied, more than half of the total nitrate leaching occurred in the amaranth-growing season in 2000. However, most of the nitrate leaching was observed in cauliflower and spinach seasons in 2001. Furthermore, the nitrate leaching in 2000 was significantly higher than that in 2001. Previous research proposed that the amount of nitrate leaching is mainly determined by the amount and intensity of rainfall [7,22]. Compared with 2001, rainfall was greater and more intense in 2000, and most of it occurred during the amaranth season in this study (Figure 6).

Previous research reported that less irrigation and drip irrigation could significantly increase N residual by saving water input [52,53,54]. In this study, although drip irrigation was not employed, water input declined by about 30% under the optimized irrigation treatment (W2). The amount of N residual in each vegetable-growing season varied with different irrigation and fertilizer management. Except for the W2N2 treatment in 2000, the N residual in the cauliflower season was positive, and was the highest among the three vegetables. This was related to the higher N application in this region [55,56]. During the amaranth-growing season, the N residual was negative under most conditions, especially under the optimized fertilizer treatments. Our results indicated that amaranth N uptake ranged from 33.2 to 64.4 kg N ha^−1^ under the optimized fertilizer management (Table 4 and Table 5) and could surpass the N input in the amaranth season. This means amaranth growth possibly consumed a part of residual N from the cauliflower season under the optimized fertilizer treatments.

### 3.3. Vegetable Yield, WUE, and NUE Responses to Different Water and N Management Practices

As is known, reduced irrigation and nutrient input mostly decreases the yield in most of agricultural systems; however, it is not applicable to regions with excessive water and nitrogen input [57,58]. Similarly, as shown in Table 2 and Table 3, the total yield of three vegetables generally follows the trend that the yield decreased with the decrease in water and N inputs, but there is no significant variation among the different irrigation and fertilizer treatments in both years. However, there was a large variation in the WUEs and NUEs of different treatments. 

Previous studies confirmed that WUE had a negative relationship with irrigation input [10,59,60], which is consistent with our results. Conversely, investigations into field crops indicated that increasing nitrogen application can enhance the WUE in wheat due to enhanced root development [61]. However, this effect was not pronounced in our current study, which may be attributed to the less-developed root systems typically associated with vegetable crops.

The simulation results indicated that for the same irrigation management, optimizing the fertilizer treatments increased the annual average NUEs. Furthermore, the optimization of irrigation practices alone also significantly enhanced the annual average NUEs, corroborating the findings from prior research [52]. Our results were significantly higher than those reported by Wang et al. [7], but similar to the results of other studies [49,51]. In this study, the following vegetables grown could deplete N left by pre-season vegetables under optimized fertilizer management. Therefore, the effects of this fertilizer management on improving NUE were better than the results obtained by only reducing N fertilizer input.

The results of our study indicate that excess N or water does not significantly improve the yield of the vegetables, as it is mainly limited by the genetic potential of vegetables, and the less-developed root system of vegetable crops allows a large amount of water and N to be wasted [7,8]. However, the judicious timing of water and nitrogen applications can markedly improve the efficiency of resource utilization in the vegetable production system [62,63].

## 4. Materials and Methods

### 4.1. Site Description

The field experiment was conducted at the China Agricultural University experiment station, located in Dongbeiwang Town, Beijing, China (39.9° N, 116.4° E). The site belongs to a temperate semi-humid continental monsoon climate. The annual average air temperature and precipitation are 11.5 °C and 630 mm. The soil type is Calcaric Cambisol [64]. In the topsoil, soil organic matter is 15 g kg^−1^, total N is 1.2 g kg^−1^, and the available phosphorus and available potassium are 21.9 mg kg^−1^ and 109.6 mg kg^−1^, respectively.

### 4.2. Experimental Design and Field Management

Vegetable systems: The field experiment lasted for two years, from April 2000 to October 2001. Based on the climate and physiological characteristics of vegetables, three types of vegetables were cultivated each year, including cauliflower (*Brassica oleracea* L. var. botrytis cv. Xuefeng), amaranth (*Amaranthus tricolor* L. Local variety) and spinach (*Spinacia oleracea* L. cv. Boza 18) (Figure S3). The detailed planting and harvest dates of the three vegetables in both years are shown in Table 6. Cauliflower was planted with 50 cm between plants and 42.5 cm between rows. Amaranth and spinach were both sown in strips with 24.0 cm between rows.

Water and fertilization practices: There were two water treatments (W1, traditional irrigation; W2, optimal irrigation) and three N fertilizer treatments (N1, traditional N fertilizer; N2, optimal N fertilizer; N3, 80% N2) with three replications in this study. A total of six treatments (W1N1, W1N2, W1N3, W2N1, W2N2, and W2N3) were arranged in a randomized block design, with the area of each plot being 12 m × 12 m. 

The traditional water (W1) treatment was based on local farmers’ practices. For the W2 treatment, the soil water content was kept at 50%-80% of field capacity. Time Domain Reflectometry (TDR) probes were installed to monitor the soil water content and calculate the irrigation amount needed in W2. The three N treatments were implemented as follows: (1) N1: same as farmers’ practice; (2) N2: the rate of optimal N was calculated by the Expert-N system (see Equation (1)) [65]; (3) based on the N2 treatment, the rate for N fertilizer of spinach was reduced by about 20%.

N_opt_ = N_upt_ + N_res_ + N_loss_ − N_ini_ − N_hum_ − N_root_ (1)

where N_opt_ is the optimal N fertilizer dosage; N_upt_ is the expected amount of N taken up by the crop during the whole growing season; N_res_ is the amount of mineral N in the root zone after harvest; N_loss_ is the amount of N loss calculated with the following equation: N_loss_ = N_input_ × 0.019 × growing season (week), N_input_ refers to the quantity of nitrogen utilized in farmers’ practices; N_ini_ is the amount of mineral N in the root zone at the beginning of each crop growing season; N_hum_ is the amount of N from soil organic N mineralization; and N_root_ is the amount of N from crop residue mineralization. In this experiment, most of the crop residues were removed from the field, so this part was zero.

In all the treatments, manure was applied as base fertilizer and mixed with tillage into the topsoil 20 cm before cauliflower cultivation each year. The amount of manure was 85 and 120 kg N ha^−1^ in 2000 and 2001, respectively. Under the W1 and W2 treatments, the methods of irrigation were furrow and micro-sprinkler irrigation systems, respectively. For cauliflower, N fertilizer was topdressed at the end of April and mid-May each year. Amaranth did not receive any topdressing. For spinach, N fertilizer was topdressed in mid-September and early October each year. For each vegetable season, except for amaranth, the rate of fertilizer N application each time was one-third of the total amount. Urea was applied by broadcasting, followed by immediate irrigation. The amount of phosphorus and potassium were the same for all the treatments. Conventional tillage was applied before each vegetable sowing. The details of water and N management practices are shown in Table S1. 

### 4.3. Soil and Crop Sampling and Analyzing

We excavated a soil profile pit to 120 cm, and soil samples were collected from four soil layers: 0–30, 30–60, 60–90, and 90–120 cm before the experiment. Basic soil physical/chemical properties, including soil bulk density, soil saturated water content, field capacity, wilting point, and pH, were measured. The soil bulk density and water content were measured by oven-drying for 24 h at 105 °C [66]. The soil water characteristic curves for estimating the saturated water content, *θ_s_*; field capacity, *θ_fc_*; and wilting water content, *θ_wp_* were determined with a pressure plate apparatus at 0, −33 and −1500 kPa, respectively [67]. Soil pH was determined using a soil/water (1:2.5) slurry. The results are shown in Table 7.

In each plot, the soil volumetric water content from 0 to 120 cm at 30 cm intervals in the soil profile was measured by TDR (MP-917) every two days. The experiment initially aimed to investigate nitrate leaching at a 90 cm depth under various water and fertilizer regimes. Therefore, the suction cups were installed at only 90 cm soil depth. Soil solutions were periodically collected at intervals ranging from 7 to 15 days, and were analyzed for mineral N to ascertain soil nitrate concentrations with the continuous flow analyzer (TRAACS 2000). Plant biomass, fresh yield, and nitrogen content were quantified on a weekly basis through the collection of plant samples. The yields of vegetables were determined within a 1 m^2^ quadrat, replicated four times within each experimental plot.

### 4.4. Model Description and Input

In this study, the EU-Rotate_N model was utilized to simulate soil water movement, nitrogen transport, and vegetable growth. It contains modules for simulating various soil/plant processes including root development, N mineralization from soil organic matter and crop residues, water and N uptake by roots, and water movement and N transport in soil. The organization of the main model modules is shown in Figure S4. In the EU-Rotate_N model, the vegetable growth algorithm was derived from the N_ABLE model [68,69]. Water demand for crop growth was calculated by the Penman/Monteith method of the FAO [37], while soil water movement was simulated following Ritchie’s soil water balance approach [70]. Surface runoff estimation adhered to the methodology advocated by the U.S. National Resource Conservation Service [71]. The N mineralization simulation was based on the DAISY model’s algorithm [72]. Ammonia emissions from manure were quantified using the empirical formula from the ALFAM model [73]. The simulations of urea hydrolysis and gaseous N emissions were based on the AMOVOL model [74]. A detailed model description is available in the literature [24,68].

The model inputs include site location (latitude and altitude), basic soil physico-chemical properties (e.g., bulk density and hydraulic properties), crop information (species, row spacing, planting density, and sowing and harvesting schedules), field management practices, soil initial water and mineral nitrogen condition, and local meteorological data [22,24,28].

### 4.5. Model Evaluation Statistics and Data Analysis

Three statistical indices were used to evaluate the model performance: root mean square error (*RMSE*), Nash/Sutcliffe efficiency coefficient (*E*), and agreement index (*d*) [32,75].

(2) $$RMSE=\sqrt{\sum_{i=1}^{n}\frac{{\left(P_{i}-O_{i}\right)}^{2}}{n}}$$

(3) $$E=1-\frac{\sum_{i=1}^{n}{\left(O_{i}-P_{i}\right)}^{2}}{\sum_{i=1}^{n}{\left(O_{i}-O\right)}^{2}}$$

(4) $$d=1-\frac{\sum_{i=1}^{n}{\left(O_{i}-P_{i}\right)}^{2}}{\sum_{i=1}^{n}{\left(|P_{i}-O|+|O_{i}-O|\right)}^{2}}$$

where *n* is the number of samples, *P_i_* and *O_i_* are the simulated and observed values, and *O* is the mean of the measured data. The closer the value of root mean square error (*RMSE*) is to 0, the more accurate the model. Modeling efficiency (*E*) ranges from −∞ to 1. The agreement index (*d*) represents the ratio of the mean square error and the potential error. When *d* = 1, it indicates a perfect simulation of results. Based on van Liew and Garbrecht [31], an acceptable simulation should have *E* > 0.36 and *d* > 0.7.

The analysis of variance (ANOVA)was used to compare the effects of the different water and N management on vegetable yields using SPSS 23 (International Business Machines Corporation, Armonk, NY, USA). The graphs were generated using Origin 2018 (OriginLab Corporation, Northampton, MA, USA). 

## 5. Conclusions

In this study, we used the soil water content, nitrate concentration, crop dry matter, and other data collected from an open-field vegetable field with different irrigation and N fertilizer managements to validate the EU-Rotate_N model. Statistical analysis indicated that the EU-Rotate_N model could simulate the dynamics of water and N in an open-field intensive vegetable system in the North China Plain.

The results showed the yields of amaranth and spinach had no significant differences among all the treatments in 2000 and 2001. However, the cauliflower yield under the W1N2 and W1N3 treatments obviously reduced in 2001, indicating that excess fertilizer N or water does not significantly improve the yield of vegetables. 

Unlike greenhouse vegetable fields, open-field vegetable production had higher gaseous N losses than nitrate leaching, which was the main form of N loss under all the treatments in this study. Gaseous N loss accounted for 16.5% of the total N input, while the annual amount of nitrate leaching was less and accounted for only 8.4% of the total N input. Optimizing irrigation and N fertilizer practices could effectively reduce gaseous N release and nitrate leaching, and increase NUE. Compared with W1, gaseous N loss under W2 was reduced by 18–26% and the annual average NUEs increased by 22–29%. Compared to the N1 treatments, gaseous N under the N2 and N3 treatments reduced by 48–72% and increased NUE by 17–37%. Based on two years of field experiments, the simulation results showed that the W2N3 treatment was the best water and N fertilizer management, and should be recommended to local farmers. 

Open-field vegetable production is significantly influenced by climatic conditions. In 2000, the rainfall reached 151 mm during the period of amaranth, which led to the highest drainage and nitrate leaching. In contrast, it was only 69.1 mm in 2001, resulting in no drainage or nitrate leaching. Climate change will intensify water scarcity, making it necessary to apply greenhouse cultivation techniques for vegetable crops that require sufficient water supply, especially in regions with low rainfall. Additionally, farmers can adopt drought-tolerant vegetable crops such as amaranth or spinach instead of cauliflower especially in arid areas to reduce irrigation costs and drought risks. Furthermore, rationally adjusting the cropping structure can allow subsequent crops to fully utilize the residual N in the soil, thereby reducing the risk of environmental pollution. 

## Figures and Tables

**Table 1:** Statistical indices for the predicted soil water content and nitrate concentration at different depths for the validation treatments (W1N2, W1N3, W2N1, W2N2, and W2N3).

	Soil Layer (cm)	RMSE	E	d
Soil water content	0–15	0.04	0.38	0.83
	15–30	0.02	0.47	0.87
	30–60	0.01	0.46	0.88
	60–90	0.01	0.86	0.97
	90–120	0.01	0.92	0.98
Soil NO_3_-N concentration	60–90	13.41	0.87	0.97

Note: RMSE, root mean square error; E, Nash/Sutcliffe efficiency coefficient; d, agreement index.

**Table 2:** Yield and soil water balance in 0.9 m soil profile under three vegetable continuous cropping systems with different water and fertilizer treatments in 2000.

Treatments	Vegetable	RF mm	I mm	ET mm	D mm	Y kg ha^−1^	WUE kg m^−3^	IWUE kg m^−3^	Wbalance mm
W1N1	cauliflower	45.1	294.1	327.8	20.4	23,400 a	7.1	8.0	−9.0
	amaranth	151.0	85.5	104.0	111.5	14,300 a	13.8	16.7	21.0
	spinach	65.6	128.2	130.2	34.6	34,800 a	26.7	27.1	29.0
	total	261.7	507.8	562.0	166.5	72,500	12.9	14.3	41.0
W1N2	cauliflower	45.1	294.1	323.1	20.7	19,600 ab	6.1	6.7	−4.6
	amaranth	151.0	85.5	99.4	108.7	13,700 a	13.8	16.0	28.4
	spinach	65.6	128.2	128.5	37.1	32,900 a	25.6	25.7	28.2
	total	261.7	507.8	551.0	166.5	66,200	12.0	13.0	52.0
W1N3	cauliflower	45.1	294.1	319.2	19.5	20,500 ab	6.4	7.0	0.5
	amaranth	151.0	85.5	95.8	108.6	10,800 a	11.3	12.6	32.1
	spinach	65.6	128.2	127.7	40.2	36,400 a	28.5	28.4	25.9
	total	261.7	507.8	542.7	168.3	67,700	12.5	13.3	58.5
W2N1	cauliflower	45.1	208.5	281.2	2.9	21,400 ab	7.6	10.3	−30.5
	amaranth	151.0	68.3	100.9	35.8	13,100 a	13.0	19.2	82.6
	spinach	65.6	89.3	114.8	4.0	33,100 a	28.8	37.1	36.1
	total	261.7	366.1	496.9	42.7	67,600	13.6	18.5	88.2
W2N2	cauliflower	45.1	208.5	273.3	1.2	19,700 ab	7.2	9.4	−20.9
	amaranth	151.0	68.3	100.4	38.0	15,200 a	15.1	22.3	80.9
	spinach	65.6	89.3	115.3	4.1	32,500 a	28.2	36.4	35.5
	annual	261.7	366.1	489.0	43.3	67,400	13.8	18.4	95.5
W2N3	cauliflower	45.1	208.5	284.8	0.1	18,100 bc	6.4	8.9	−31.3
	amaranth	151.0	68.3	101.1	30.7	16,500 a	16.3	24.2	87.5
	spinach	65.6	89.3	115.4	6.8	33,900 a	29.4	38.0	32.7
	total	261.7	366.1	501.3	37.6	68,500	13.7	18.7	88.9

Note: RF, rainfall. I, irrigation. ET, evapotranspiration. D, water drainage. Y, fresh yield. WUE (water use efficiency) = Y/ET. IWUE (irrigation water use efficiency) = Y/I. Wbalance = RF + I − ET − D. Different letters indicate significant differences in yield for same vegetable under different treatments at *p* < 0.05.

**Table 3:** Yield and soil water balance in 0.9 m soil profile under three vegetable continuous cropping systems with different water and fertilizer treatments in 2001.

Treatments	Vegetable	RF mm	I mm	ET mm	D mm	Y kg ha^−1^	WUE kg m^−3^	IWUE kg m^−3^	Wbalance mm
W1N1	cauliflower	21.9	290.7	310.5	30.5	19,200 a	6.2	6.6	−28.4
	amaranth	69.1	107.4	127.7	0.0	22,500 a	17.6	20.9	48.8
	spinach	52.7	123.4	121.3	24.4	30,100 a	24.8	24.4	30.4
	total	143.7	521.5	559.5	54.9	71,800	12.8	13.8	50.8
W1N2	cauliflower	21.9	290.7	304.3	31.0	16,200 b	5.3	5.6	−22.7
	amaranth	69.1	107.4	129.6	0.0	18,600 a	14.4	17.3	46.9
	spinach	52.7	123.4	118.0	16.8	30,200 a	25.6	24.5	41.3
	total	143.7	521.5	551.9	47.8	65,000	11.8	12.5	65.5
W1N3	cauliflower	21.9	290.7	297.1	29.5	14,500 c	4.9	5.0	−14.0
	amaranth	69.1	107.4	128.1	0.0	18,300 a	14.3	17.0	48.4
	spinach	52.7	123.4	118.0	26.9	31,400 a	26.6	15.4	31.2
	total	143.7	521.5	543.2	56.4	64,200	11.8	12.3	65.6
W2N1	cauliflower	21.9	207.6	258.0	0.9	19,300 a	7.5	9.3	−29.4
	amaranth	69.1	74.0	112.7	0.0	22,400 a	19.9	30.3	30.4
	spinach	52.7	84.6	117.3	0.4	31,600 a	26.9	37.4	19.6
	total	143.7	366.2	488.0	1.3	73,300	15.0	20.0	20.6
W2N2	cauliflower	21.9	207.6	257.6	0.2	19,000 a	7.4	9.2	−28.3
	amaranth	69.1	74.0	114.0	0.0	21,200 a	18.6	28.6	29.1
	spinach	52.7	84.6	118.9	1.7	25,900 a	21.8	30.6	16.7
	annual	143.7	366.2	490.5	1.9	66,100	13.5	18.1	17.5
W2N3	cauliflower	21.9	207.6	254.9	0.6	18,600 a	7.3	9.0	−26.0
	amaranth	69.1	74.0	113.7	0.0	15,200 a	13.4	20.5	29.4
	spinach	52.7	84.6	117.6	9.5	30,400 a	25.9	35.9	10.2
	total	143.7	366.2	486.2	10.1	64,200	13.2	17.5	13.6

Note: RF, rainfall. I, irrigation. ET, evapotranspiration. D, water drainage. Y, fresh yield. WUE (water use efficiency) = Y/ET. IWUE (irrigation water use efficiency) = Y/I. Wbalance = RF + I − ET − D. Different letters indicate significant differences in yield for same vegetable under different treatments at *p* < 0.05.

**Table 4:** N balance in 0.9 m soil profile under three vegetable continuous cropping systems with different water and fertilizer treatments in 2000.

Treatments		Manu	Fert	Nirr	Nnet	Nup	Ngas	Nlea	NUE kg kg^−1^	Nbalance kg N ha^−1^
		kg N ha^−1^								
W1N1	cauliflower	75	450.0	11.5	25.0	162.5	141.3	7.8	75.1	249.9
	amaranth	0	100.0	3.3	23.5	46.9	24.9	129.2	71.1	−74.2
	spinach	0	309.0	5.0	32.2	120.0	46.7	58.7	154.4	120.8
	total	75	859.0	19.8	80.7	329.4	212.9	195.7	98.2	296.5
W1N2	cauliflower	75	165.9	11.5	24.5	158.6	65.8	6.9	84.7	45.6
	amaranth	0	26.0	3.3	21.6	44.1	12.7	79.0	100.9	−84.9
	spinach	0	81.4	5.0	32.5	113.7	16.1	29.4	206.7	−40.3
	total	75	273.3	19.8	78.6	316.4	94.6	115.3	125.8	−79.6
W1N3	cauliflower	75	189.8	11.5	24.6	163.6	79.2	6.3	82.3	51.8
	amaranth	0	26.2	3.3	21.4	33.2	14.6	88.9	79.0	−85.8
	spinach	0	72.0	5.0	32.0	117.7	16.0	32.4	219.1	−57.1
	total	75	288.0	19.8	78.0	314.5	109.8	127.6	122.7	−91.1
W2N1	cauliflower	75	450.0	8.1	24.0	159.8	116.4	0.8	77.3	280.1
	amaranth	0	100.0	2.7	28.7	44.9	20.1	22.0	150.6	44.4
	spinach	0	309.0	3.5	32.7	122.9	38.5	6.6	197.0	177.2
	total	75	859.0	14.3	85.4	327.6	175.0	29.4	127.1	501.7
W2N2	cauliflower	75	83.6	8.1	24.1	165.3	32.0	0.3	99.7	−6.8
	amaranth	0	25.7	2.7	28.3	49.6	15.2	15.5	189.3	−23.6
	spinach	0	83.9	3.5	34.1	118.3	12.2	3.3	242.9	−12.3
	total	75	193.2	14.3	86.5	333.2	59.4	19.1	163.7	−42.7
W2N3	cauliflower	75	101.7	8.1	23.8	164.7	36.4	0.0	90.0	7.5
	amaranth	0	31.5	2.7	28.2	57.1	9.0	8.2	222.1	−11.9
	spinach	0	55.4	3.5	32.7	116.3	8.1	4.0	264.0	−36.8
	total	75	188.6	14.3	84.7	338.1	53.5	12.2	169.6	−41.2

Note: Manu, manure; Fert, fertilizer; Nnet, net mineralization; Nirr, N input by irrigation; Nup, crop N uptake. Nlea, nitrate leaching. Ngas, gaseous N emission. NUE (nitrogen use efficiency) = Fresh yield/(Nup + Nlea + Ngas). Nbalance = N input − N output.

**Table 5:** N balance in 0.9 m soil profile under three vegetable continuous cropping systems with different water and fertilizer treatments in 2001.

Treatments		Manu	Fert	Nirr	Nnet	Nup	Ngas	Nlea	NUE kg kg^−1^	Nbalance kg N ha^−1^
		kg N ha^−1^								
W1N1	cauliflower	120	450.0	11.3	32.8	156.8	113.3	26.6	64.7	317.4
	amaranth	0	100.0	4.2	33.9	61.6	16.0	0.0	289.9	60.5
	spinach	0	309.0	4.8	36.0	104.2	49.9	30.4	163.1	165.3
	total	120	859.0	20.3	102.7	322.6	179.2	57.0	128.5	543.2
W1N2	cauliflower	120	109.5	11.3	33.5	135.8	44.0	13.8	83.7	80.7
	amaranth	0	0.0	4.2	34.6	55.6	7.0	0.0	297.1	−23.8
	spinach	0	126.3	4.8	35.0	102.9	22.9	8.4	225.0	31.9
	total	120	235.8	20.3	103.1	294.3	73.9	22.2	166.5	88.8
W1N3	cauliflower	120	71.6	11.3	29.4	122.6	34.4	8.2	87.8	67.1
	amaranth	0	0.0	4.2	34.0	53.0	8.5	0.0	297.6	−23.3
	spinach	0	99.6	4.8	35.4	102.5	19.3	15.2	229.2	2.8
	total	120	171.2	20.3	98.8	278.1	62.2	23.4	176.5	46.6
W2N1	cauliflower	120	450.0	8.1	30.5	156.6	81.9	1.1	80.6	369.0
	amaranth	0	100.0	2.9	33.7	62.4	19.5	0.0	273.5	54.7
	spinach	0	309.0	3.3	34.0	112.9	32.1	0.6	217.0	200.7
	total	120	859.0	14.3	98.2	331.9	133.5	1.7	156.9	624.4
W2N2	cauliflower	120	62.0	8.1	30.5	141.1	22.2	0.0	116.4	57.3
	amaranth	0	0.0	2.9	33.2	64.4	3.2	0.0	313.6	−31.5
	spinach	0	98.9	3.3	35.0	108.1	12.1	0.5	214.6	16.5
	total	120	160.9	14.3	98.7	313.6	37.5	0.5	188.0	42.3
W2N3	cauliflower	120	68.5	8.1	29.4	139.8	25.7	0.2	112.3	60.3
	amaranth	0	0.0	2.9	33.1	51.8	4.5	0.0	270.0	−20.3
	spinach	0	71.8	3.3	34.5	112.3	10.6	3.5	240.5	−16.8
	total	120	140.3	14.3	97.0	303.9	40.8	3.7	184.3	23.2

Note: Manu, manure; Fert, fertilizer; Nnet, net mineralization; Nirr, N input by irrigation; Nup, crop N uptake. Nlea, nitrate leaching. Ngas, gaseous N emission. NUE (nitrogen use efficiency) = Fresh yield/(Nup + Nlea + Ngas). Nbalance = N input − N output.

**Table 6:** Growth periods of different vegetables in 2000 and 2001.

	Cauliflower		Amaranth		Spinach	
Year	Sowing Date	Harvest Date	Sowing Date	Harvest Date	Sowing Date	Harvest Date
2000	3 April	6 June	1 July	30 July	4 September	14 October
2001	13 April	6 June	24 June	22 July	6 September	29 October

**Table 7:** Soil physical and chemical properties in 0-120 cm soil profile.

Soil Layer (cm)	Particle Fraction (%)			Soil Texture	BD (g cm^−3^)	*θ_fc_* (cm^3^ cm^−3^)	*θ_wp_* (cm^3^ cm^−3^)	*θ_s_* (cm^3^ cm^−3^)	pH
	Sand	Silt	Clay						
0–30	32.7	50.2	17.1	Loam	1.33	0.33	0.14	0.50	7.9
30–60	16.4	56.8	26.8	Silt loam	1.52	0.35	0.15	0.43	7.9
60–90	30.7	47.6	21.7	Loam	1.43	0.35	0.14	0.44	8.0
90–120	35.0	46.4	18.6	Loam	1.62	0.33	0.14	0.43	8.0

Note: BD, bulk density; *θ_s_*, saturated water content; *θ_fc_*, field capacity; *θ_wp_*, wilting water content.

## Data Availability

The data is contained within the manuscript.

## Supplementary Materials

The following supporting information can be downloaded at: https://www.mdpi.com/article/10.3390/plants13152150/s1, Table S1. Application of irrigation and N fertilizer for field vegetable cultivation in 2000 and 2001. Table S2. Crop and N transformation parameters used in EU-Rotate_N model. Figure S1. Comparisons of simulated and measured crop dry matter weight for three vegetables under different treatments in 2000. Figure S2. Comparisons of simulated and measured crop dry matter weight for three vegetables under different treatments in 2001. Figure S3. Some photos of the experiment site. Figure S4. The organization of the main modules of EU-Rotate_N model.
