# Supplementary material for: Assessing the Fates of Water and Nitrogen on an Open-Field Intensive Vegetable System under an Expert-N System with EU-Rotate_N Model in North China Plain
Source: Plants (Basel). 2024 Aug 3;13(15):2150. doi: 10.3390/plants13152150 (PMC11314188; doi:10.3390/plants13152150)
Supplement: Supplementary file 1 [file plants-13-02150-s001.zip › plants-2997937-supplementary.pdf]

Supplementary Materials

Assessing the fates of water and nitrogen on an open-field intensive vegetable system under an Expert-N system with EU-Rotate_N model in North China Plain

Yuan Sun [1,2,#], Shaoqing Chen [1,#], Puyu Feng [1], Qing Chen[3], Kelin Hu [1,*]

[1] College of Land Science and Technology, China Agricultural University; Key Laboratory of Arable Land Conservation (North China), Ministry of Agriculture and Rural Affairs, Beijing 100193, China

[2] Agricultural Information Institute of Chinese Academy of Agricultural Science, Beijing, 100081, China

[3] College of Resources and Environmental Sciences, China Agricultural University, Beijing 100193, China

# These authors contributed equally to this work

*Corresponding author:
Prof. Kelin Hu

Email: hukel@cau.edu.cn

Table S1. Application of irrigation and N fertilizer for field vegetable cultivation in 2000 and 2001

Year	Vegetable species	W1N1		W1N2		W1N3		W2N1		W2N2		W2N3	
		Irri	Nfer	Irri	Nfer	Irri	Nfer	Irri	Nfer	Irri	Nfer	Irri	Nfer
2000	Cauliflower	294.1	450.0	294.1	165.9	294.1	189.8	208.5	450.0	208.5	83.6	208.5	101.7
	Amaranth	85.8	100.0	85.8	26.0	85.8	26.2	68.3	100.0	68.3	25.7	68.3	31.5
	Spinach	128.2	309.0	128.2	81.4	128.2	72.0	89.3	309.0	89.3	83.9	89.3	55.4
2001	Cauliflower	290.7	450.0	290.7	109.5	290.7	71.6	207.6	450.0	207.6	62.0	207.6	68.5
	Amaranth	107.4	100.0	107.4	0.0	107.4	0.0	74.0	100.0	74.0	0.0	74.0	0.0
	Spinach	123.4	309.0	123.4	126.3	123.4	99.6	84.6	309.0	84.6	98.9	84.6	71.8

Note: Irri, irrigation; Nfer, N fertilizer.

Table S2. Crop and N transformation parameters used in EU-Rotate_N model.

Parameters	Description	Vegetable	Value
K_ini	Crop coefficient in initial stage	Cauliflower	0.15
		Amaranth	0.30
		Spinach	0.15
K_mid	Crop coefficient in middle stage	Cauliflower	0.95
		Amaranth	0.90
		Spinach	0.95
K_end	Crop coefficient in end stage	Cauliflower	0.85
		Amaranth	0.95
		Spinach	0.95
RLUX	Luxury N consumption coefficient	Cauliflower	1.1
		Amaranth	1.0
		Spinach	1.0
α	Empirical parameter of luxury N consumption	Cauliflower	2.5
		Amaranth	1.0
		Spinach	1.45
β	Empirical parameter of luxury N consumption	Cauliflower	0.6
		Amaranth	2.6
		Spinach	3.0
SOM_SlowDecCoeff	SOM Slow decomposition standard coefficient (d^{-1})		4.3e-5
SOM_FastDecCoeff	SOM fast decomposition standard coefficient (d^{-1})		1.4e-4
SMB_SlowMaintRate	Slow maintenance respiration coefficient of soil microbial biomass (d^{-1})		0.001
SMB_FastMaintRate	Fast maintenance respiration coefficient of soil microbial biomass (d^{-1})		0.01
SMB_Uti_Eff	Substrate of soil microbial biomass utilization efficiency (0-1)		0.6
SOM_FastUti_Eff	Substrate of soil OM fast utilization efficiency (0-1)		0.5
AOM_FastUti_Eff	Substrate of add OM utilization efficiency (0-1)		0.5
Hydrolysis_KM	Urea hydrolysis rate (d^{-1})		0.003
Nitrification_Rate	Standard coefficient of Nitrification rate		0.01
Immob_CoeffNO3	Immobilisation rate coefficient of NO_3 (d^{-1})		0.5
Immob_CoeffNH4	Immobilisation rate coefficient of NH_4 (d^{-1})		0.5
Denit_Rate	Denitrification parameter (d^{-1})		0.2
Atmospheric_Resis	Atmospheric resistance parameter (s m^{-1})		25

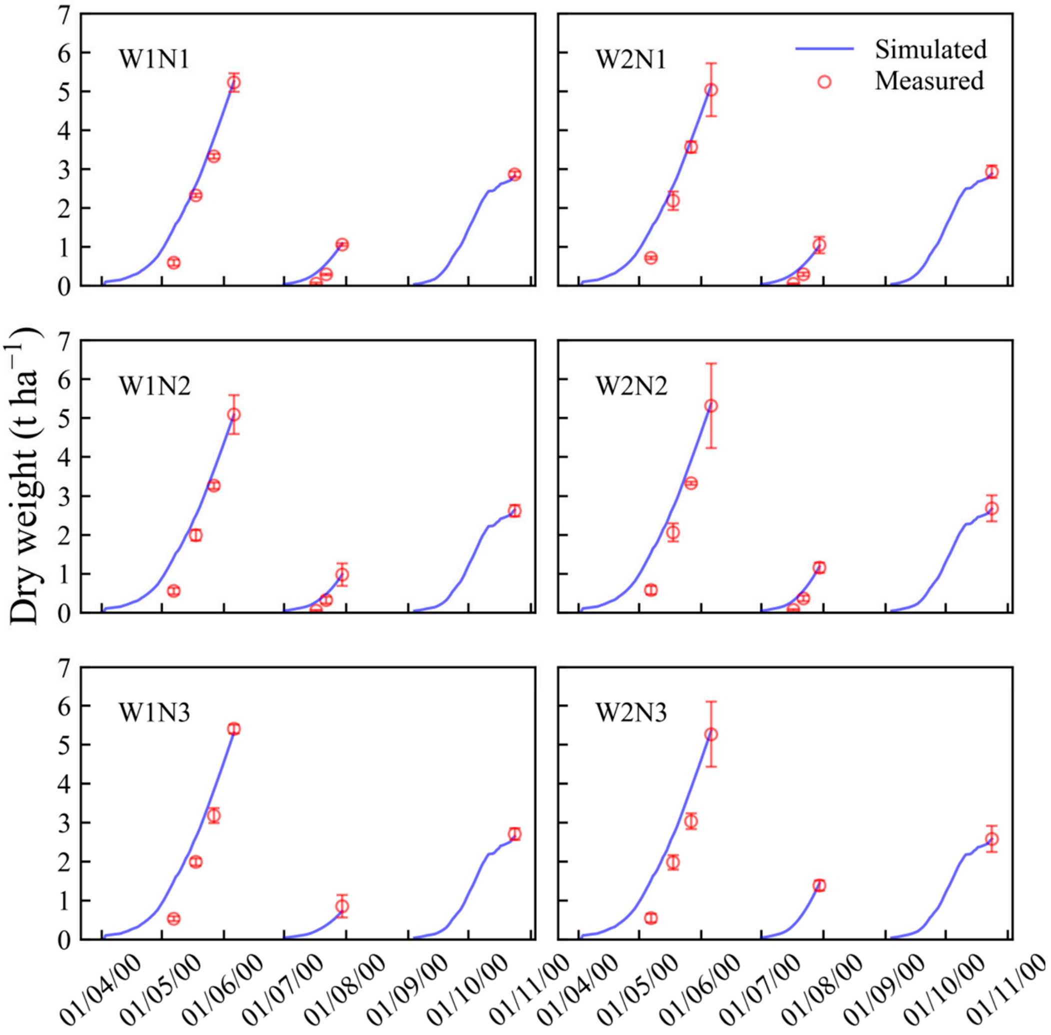

Figure S1. Comparisons of simulated and measured crop dry matter weight for three vegetables under different treatments in 2000.

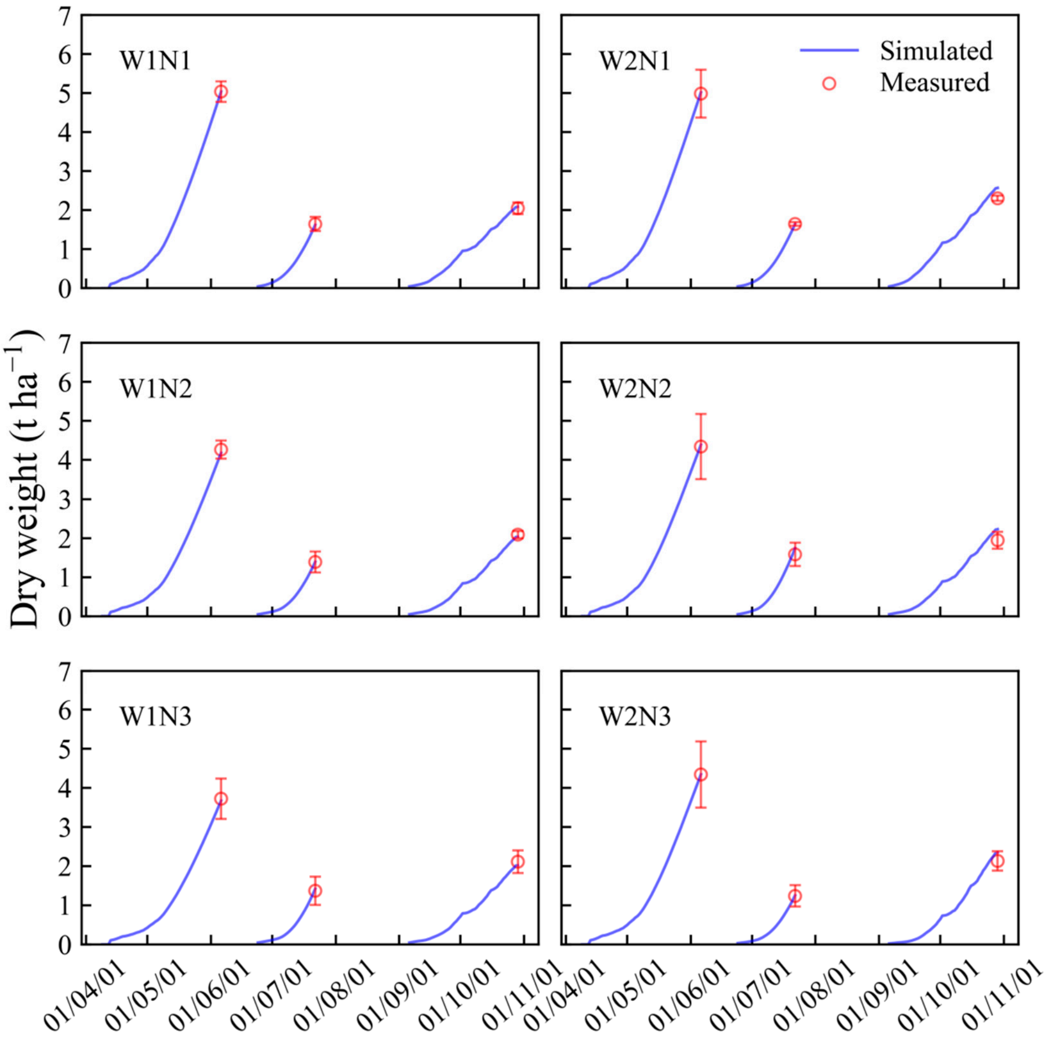

Figure S2. Comparisons of simulated and measured crop dry matter weight for three vegetables under different treatments in 2001.

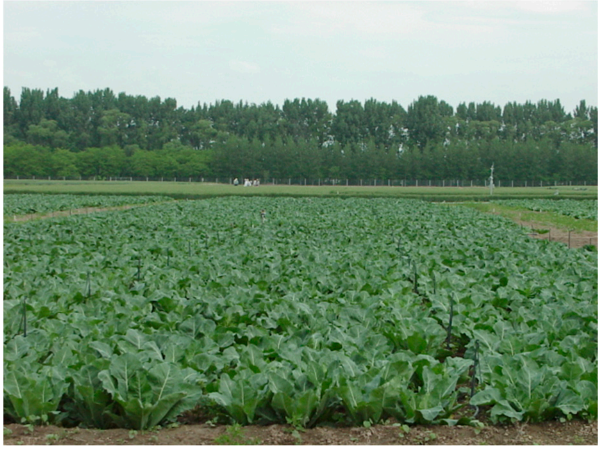

(a) Cauliflower (*Brassica oleracea L.*)

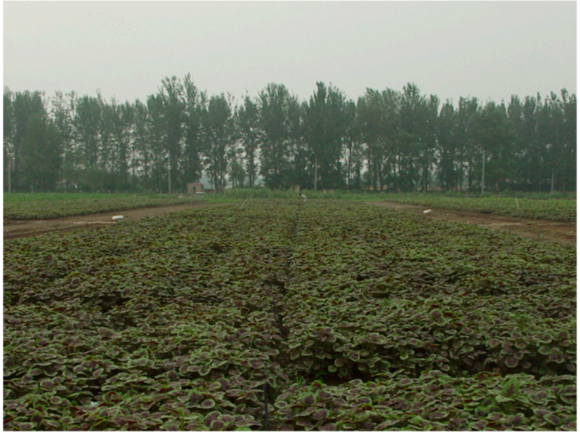

(b) Amaranth (*Amaranthus tricolor L.*)

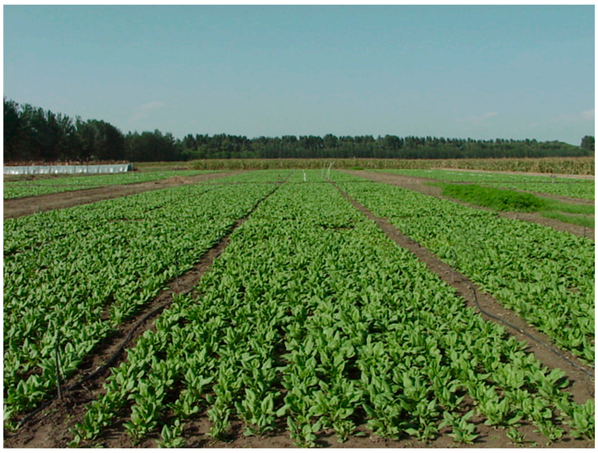

(c) Spinach (*Spinacia oleracea L.*)

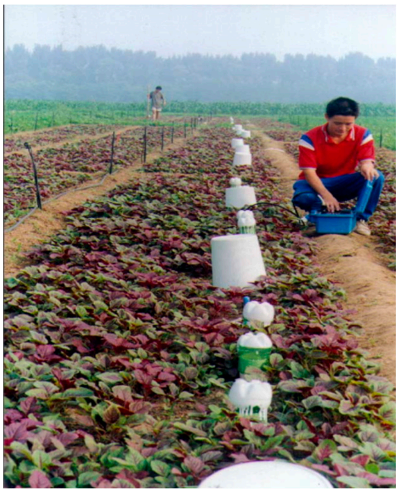

(d) Irrigation system

Figure S3. Some photos of the experiment site.

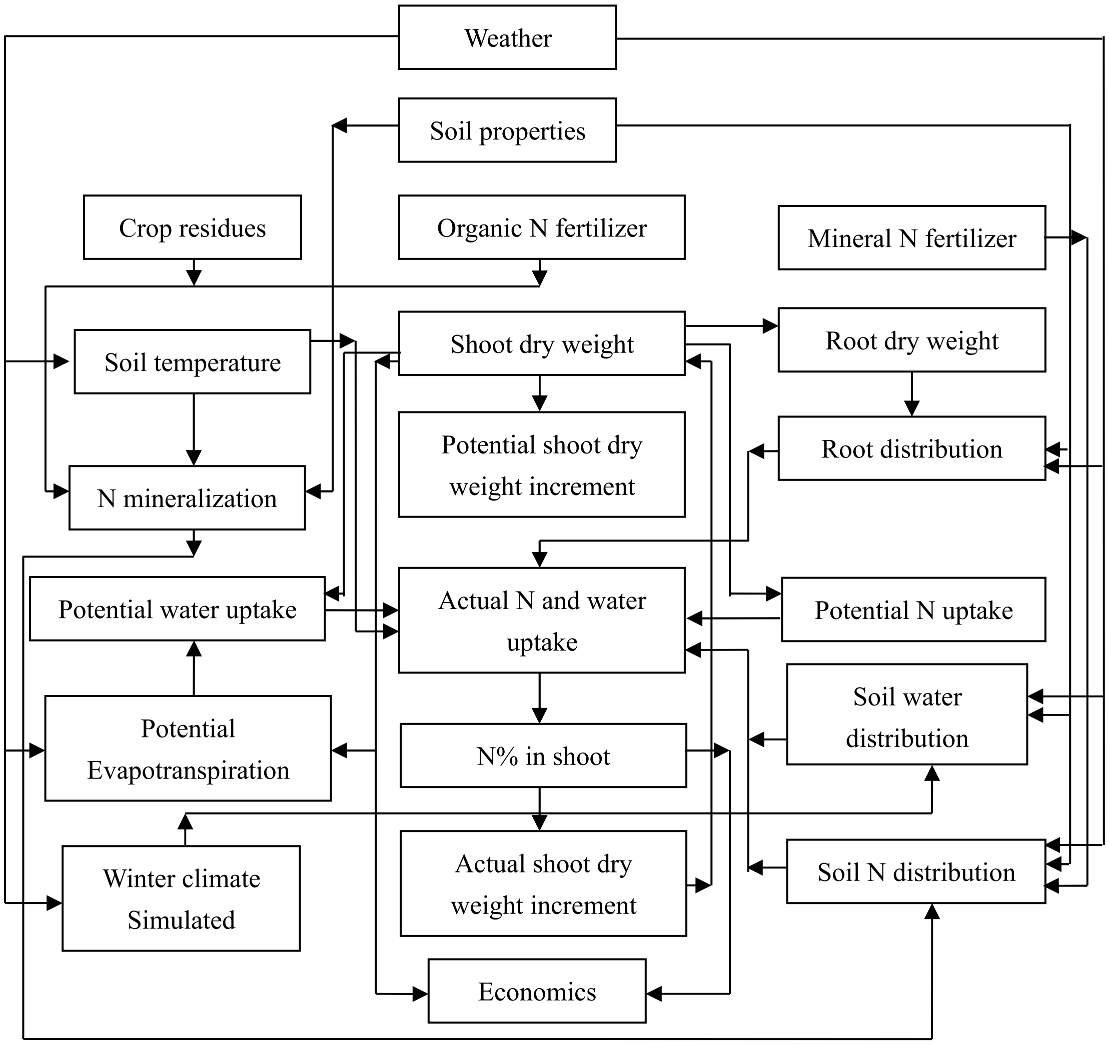

Figure S4. The organization of the main modules of EU-Rotate_N model
